# The Abbreviated Math Anxiety Scale (AMAS): Applicability and Utility in a Sample of Japanese Elementary School Children

**DOI:** 10.1002/ijop.70015

**Published:** 2025-02-11

**Authors:** Yoshifumi Ikeda, Yosuke Kita, Riko Takagi, Kento Suzuki, Irene Cristina Mammarella, Sara Caviola, Silvia Lanfranchi, Francesca Pulina, David Giofrè

**Affiliations:** ^1^ Department of Special Needs Education Tokyo Gakugei University Tokyo Japan; ^2^ Faculty of Letters Keio University Tokyo Japan; ^3^ Cognitive Brain Research Unit, Faculty of Medicine University of Helsinki Helsinki Finland; ^4^ Graduate School of Education Joetsu University of Education Niigata Japan; ^5^ Department of Developmental and Social Psychology University of Padua Padova Italy; ^6^ Disfor University of Genoa Genova Italy

**Keywords:** anxiety, elementary‐school children, general anxiety, math anxiety, mathematical achievement, mathematics assessment

## Abstract

Math anxiety negatively affects math performance and future career choices in math‐related fields. Various tools assess math anxiety, but the Abbreviated Math Anxiety Scale (AMAS) is noteworthy for its ease of administration and good psychometric properties. This study evaluates the AMAS's applicability and utility in a sample of approximately 170 Japanese elementary‐school children. Findings indicate that the Japanese version of the AMAS has very good internal consistency, good test–retest reliability and validity, both convergent and concurrent. Results from a multigroup analysis showed that the scale shows no gender bias, although girls scored higher on the AMAS despite similar performance to boys on a standardised math test. These findings highlight the AMAS's potential as a screening tool for math anxiety in young children.

## 1 Introduction

Mathematics is fundamental for success in modern society (Ancker and Kaufman 2007) and impacts various aspects of adult life, such as employment, socioeconomic status (Ritchie and Bates 2013), and even health (Reyna et al. 2009). Students with good mathematics skills have higher educational and occupational aspirations (Widlund et al. 2020), which may, in turn, influence their school choices and future professional careers (Cuder et al. 2024; Marginson et al. 2013). Specifically, students with more advanced mathematics skills are more likely to attend advanced mathematics courses and enter STEM (Science, Technology, Engineering or Mathematics) careers (Kaleva et al. 2019). Recent results confirm that higher math anxiety (MA) is related to poorer mathematical outcomes (see Caviola et al. 2022 for a recent meta‐analysis). Notably, girls tend to often exhibit higher levels of MA, which can in turn impact their mathematics performance (Caviola et al. 2017; Devine et al. 2012). For these reasons, agile and reliable instruments dedicated to the evaluation of MA are of utmost importance. The main aim of this paper was to test the applicability and utility of the Abbreviated Math Anxiety Scale (AMAS) in a sample of Japanese elementary‐school children.

MA can be generally defined as a state of discomfort caused by performing mathematical tasks. It can manifest as feelings of apprehension, tension and worry (Ashcraft and Ridley 2005), with initial signs emerging early in life (Aarnos and Perkkilä 2012) and extending into adulthood (Jameson and Fusco 2014). MA appears early in primary school, becoming more specific and progressively more detrimental throughout education (Caviola et al. 2022; Namkung et al. 2019; Pellizzoni et al. 2022). Research has shown that MA is distinct from other forms of anxiety and can impact mathematics performance beyond the effects of general anxiety and test anxiety (Mammarella, Donolato, et al. 2018; Caviola et al. 2022; Donolato et al. 2020). Higher levels of MA can lead to avoidance behaviours, where individuals with high MA may fear mathematical tasks and avoid math‐oriented university courses (Ashcraft and Moore 2009; Ashcraft and Rudig 2012). MA can also affect general cognitive abilities, interfering with working memory and impacting both math achievement (Cuder et al. 2024; Ramirez et al. 2013) and students' self‐concept (Justicia‐Galiano et al. 2017). Potential causes of MA include environmental factors, such as teachers' or schools' characteristics (Semeraro et al. 2020), and a lack of personal resources, such as resilience or self‐efficacy (Donolato et al. 2020). Japanese children, both boys and girls, perform remarkably well in math and science in international evaluations like the Programme of International Student Assessment (PISA) (OECD 2014; Schleicher 2019) and the Trends in International Mathematics and Science Study (TIMSS) (Mullis et al. 2012, 2017). However, they might also exhibit high levels of MA (Satake and Amato 1995).

It has been established that gender differences, particularly in MA, are often larger in magnitude than gender differences in mathematics. Several studies have shown that the negative relationship between MA and mathematics is stronger in girls (Devine et al. 2012; Mammarella, Caviola, and Dowker 2019). In fact, on average, girls exhibit higher levels of MA in comparison to boys (Caviola et al. 2017; Devine et al. 2012; Karimi and Venkatesan 2009; Mammarella, Caviola, and Dowker 2019), and this difference seems to increase with age (Hill et al. 2016). Intriguingly, a meta‐analysis by Else‐Quest, Hyde, and Linn (2010) showed that girls exhibited higher MA as compared to boys, despite trivial differences in mathematics between the two genders. However, more recent meta‐analyses, when considering gender as a moderator did not find differences in effect sizes (Barroso et al. 2021; Caviola et al. 2022). Most of the literature, however, is focused on Western countries (North American or European in particular), while the research is not very abundant for other countries and cultures including Asian countries for example.

Japanese children consistently perform very well in mathematics, as shown in international evaluations such as PISA and TIMSS. Results from these evaluations indicate that the performance of boys and girls in mathematics is very closely aligned (Schleicher 2019). However, despite similar mathematics performance, some findings suggest that Japanese girls exhibit higher levels of MA (Satake and Amato 1995). There have been relatively few studies investigating MA in the Japanese context. Some research has focused on university students (Fujii, 1994), while other studies have examined junior high‐school students (Kamada 1983, 1988; Sasaki 1990). Only a few studies have focused on elementary‐school children (Koenuma 2019; Watanabe and Sakuma 1998).

Several scales are available in the international literature for assessing MA. One of the most well‐established and widely known instruments for MA assessment is the Mathematics Anxiety Rating Scale (MARS) (Richardson and Suinn 1972). The good psychometric properties of the MARS prompted the development of several shorter versions for both adults and children (see Caviola et al. 2017, for a review). One of the most frequently used instruments is the AMAS (Hopko et al. 2003), which consists of nine items, making it a quick and easy‐to‐administer tool. In its original conceptualisation, the AMAS differentiates between MA levels associated with being evaluated in mathematics (math‐testing anxiety) and MA levels associated with the learning context and everyday use of mathematics (math‐learning anxiety) (Suinn and Edwards 1982). The AMAS has been adapted for children from several countries, including Croatia (Sadiković, Milovanović, and Oljača 2018), Iran (Vahedi and Farrokhi 2011), Italy (Caviola et al. 2017), Poland (Cipora et al. 2015; Szczygieł 2019), Serbia (Milovanović and Branovački 2021), Spain (Martín‐Puga et al. 2022) and the United Kingdom (Carey et al. 2017), demonstrating good psychometric properties and suitability in diverse cultures and environments.

The aim of this study was to test the utility of the AMAS in Japanese elementary‐school children. To achieve this, we first established the factorial structure of the scale. Next, we measured various forms of reliability and validity. We also performed multi‐group confirmatory factor analyses to determine if the scale was invariant across boys and girls and between younger and older children.

## 2 Methods

### 2.1 Participants

The sample initially included 173 children. Few students (*n* = 4) were absent during one or more examinations and were not included in subsequent analyses. The final sample included a total of 169 children (103 male and 66 female, 95 second‐graders [56.31% males], and 74 fifth‐graders [60.81% males]; Mage = 112.92 months [about 9 and a half years], SD = 18.06). Children were recruited through three local mainstream primary school programmes. A criterion for inclusion was the use of Japanese as a first language. Children enrolled in special needs classes were excluded. The number of children for whom parental consent was obtained is as follows: For second‐graders, 99 out of 248 (39.9%); and for fifth‐graders, 74 out of 267 (27.7%).

The order of tasks was pseudo‐randomised at the beginning and kept constant for all participants. The AMAS and J‐MAS were administered on the same day, while the Japanese Math Test was administered on a different day to avoid cognitive fatigue. The two administrations were close together. The two anxiety scales were administered in small groups, each supervised by a research assistant. The assistant checked the scale forms for any missing items before collecting them from the children. If any items were missing, the assistant asked the children to complete them. This procedure minimised the occurrence of missing data, resulting in a complete data set. Assent from the children and informed consent from their parents or legal guardians were obtained before participation. The study was conducted in accordance with the Declaration of Helsinki and approved by the Ethics Committee of Joetsu University of Education. Each child was assigned a numerical code to ensure data anonymity post‐study.

### 2.2 Measures

#### 2.2.1 Abbreviated Math Anxiety Scale

The abbreviated math anxiety scale (AMAS; Hopko et al. 2003) includes nine Likert‐type items on a 5‐point scale ranging from 1 (strongly disagree) to 5 (strongly agree). The original scale is divided into two subscales: math‐learning anxiety (*n* = 5 items) and math‐testing anxiety (*n* = 4 items). Higher scores indicate higher levels of MA. The Japanese version of the AMAS was derived from the English version using a standard translation/back‐translation process: the items were first translated into Japanese and subsequently translated back into English, with no change in meaning observed. Additionally, three independent judges evaluated the appropriateness of the Japanese translation of the scale. The adapted scale is available online (https://osf.io/nqykx/?view_only=390bc1a5966642f1bae4e262f36981a8).

#### 2.2.2 Japanese Math Anxiety Scale

The Japanese math anxiety scale (J‐MAS; Watanabe and Sakuma 1998) includes 18 Likert‐type items on a 5‐point scale ranging from 1 (strongly disagree) to 5 (strongly agree). The scale is subdivided into four subscales: math class anxiety (*n* = 9 items), math problem‐solving anxiety (*n* = 5 items), math teacher anxiety (*n* = 2 items) and math classmates' anxiety (*n* = 2 items). Higher scores indicate higher levels of MA. The reliability calculated on the current sample was excellent (Cronbach's *α* = 0.93; McDonald's *ω* = 0.90).

#### 2.2.3 Japanese Math Test

The math subtest of the Kyokenshiki Criterion‐Referenced Test‐II (Tatsuno and Kitao 2018) is a valid, reliable and standardised academic assessment commonly used in Japan. The subtest includes multiple‐choice questions, fill‐in‐the‐blank questions, short answer questions and drawing questions covering various topics such as number sense, number facts, calculation, mathematical reasoning, shape and geometric measurement. It provides scores in three areas: “mathematical thinking,” “skills in quantities and shapes” and “knowledge and understanding of quantities and shapes,” which correspond to the national curriculum standard for elementary school designed by the Ministry of Education, Culture, Sports, Science and Technology of Japan.

Since the testing was performed at the beginning of the year, we used the test for the previous grade. Thus, second‐graders took the math subtest for the first‐grade curriculum, and fifth‐graders took the subtest for the fourth‐grade curriculum. This decision ensured that the children had adequate instruction to solve the test, which would have been extremely difficult had we used the standardised test for the subsequent year. The test was administered with a time limit of 40 min and scored according to the manual. Scores were standardised for each individual assessment, with higher scores indicating higher achievement in math. Using different versions of the test, an equivalent to Cronbach's alpha coefficient was calculated via confirmatory factor analysis (CFA), rather than single items, and was found to be good (*α* = 0.81).

### 2.3 Statistical Analyses

The R program (R Core Team 2022) with the “lavaan” package (Rosseel 2012) was used. Model fit was assessed using various indices according to the criteria suggested by Hu and Bentler (1998). Since the test included ordinal values (answers at the scales were provided in Likert formats), we used the ordered function in lavaan, which provides correlations for ordinal variables, add thresholds and the mean structure to the model, set standard error to robust, and report scaled statistics to account for non‐normality in the data, when possible. Several statistics were reported, the chi‐square (*χ* ^2^), the comparative fit index (CFI), Tucker–Lewis Index (TLI) and the root mean square error of approximation (RMSEA) and the Standardised Root Mean Square Residual (SRMR). Chi‐square difference test (*Δχ* ^2^) was used for testing the difference between alternative models. It is worth noting that the robust difference test is a function of two standard statistics and is calculated using standard (not robust) chi‐square values. Based on widely accepted guidelines in structural equation modelling, a CFI and TLI value greater than 0.95 indicates a good fit, while values greater than 0.90 are considered acceptable (Hu and Bentler 1998). For RMSEA, values below 0.06 signify a good fit, and values up to 0.08 are deemed acceptable (Browne and Cudeck, 1993; Steiger 1980). Similarly, an SRMR value less than 0.08 indicates a good fit, with values up to 0.10 being acceptable (Hu and Bentler 1998). Criteria for establishing the best fitting model in MGCFA is also usually performed comparting the delta CFI for comparing alternative models with values within 0.01 considered acceptable (Hirschfeld & Von Brachel, 2014). The chi‐square difference test (*Δχ* ^2^) was used for testing the difference between nested models.

Confirmatory factor analysis was used to ascertain the number of dimensions of the AMAS. A single‐factor model was compared to a two‐factor model (including math‐learning anxiety and math‐testing anxiety). Once the factorial structure was established, we proceeded to calculate the reliability. We provided two indicators for internal consistency: Cronbach's alpha and ordered alpha (see Zumbo, Gadermann, and Zeisser 2007 for the rationale). Polyserial correlations, which are calculated in the presence of a continuous variable (i.e., the total score) and ordinal values (i.e., ordinal items), were also reported. We used the polycor package (Fox and Dusa 2019) in *R* for these calculations. Polyserial correlations are particularly useful in this context because they allow us to examine the relationship between a continuous total score and ordinal item responses, which is appropriate given the nature of our data.

The validity of the scale was assessed using different approaches: convergent validity (using the J‐MAS) and concurrent validity (predicting the performance on the Japanese math test). We then compared the concurrent validity of the AMAS against the J‐MAS, using Steiger's (1980) *Z* formula for dependent correlations.

In a subsequent step, the invariance between boys and girls and younger and older children was assessed using a multi‐group confirmatory factor analysis (MGCFA) approach. This check was necessary to ascertain potential biases in the scale. For the MGCFA, we used ordered data with the DWLS estimator. To determine various degrees of factorial invariance, we followed the criteria generally recommended by the literature on ordered data (Hirschfeld & Von Brachel, 2014). Unlike continuous data, where stricter forms of invariance can be established, the steps for factorial invariance with ordered data typically include: (1) Basic structure invariance, where the same structure is established in both groups; (2) Weak invariance, where the same loadings are established and (3) Strong invariance, where the same thresholds are established for both groups.

## 3 Results

Table 1 shows descriptive statistics for each item and polyserial correlations.

**TABLE 1:** Descriptive statistics and polyserial correlations of the AMAS.

Item	*M*	SD	Median	Min	Max	Skew	Kurtosis	*r*
1	1.96	1.32	1	1	5	1.18	0.05	0.61
2	2.02	1.23	2	1	5	0.92	−0.37	0.57
3	2.21	1.23	2	1	5	0.73	−0.40	0.61
4	1.95	1.26	1	1	5	1.15	0.15	0.64
5	2.96	1.51	3	1	5	0.05	−1.48	0.66
6	2.44	1.41	2	1	5	0.42	−1.22	0.49
7	1.72	1.11	1	1	5	1.44	1.05	0.54
8	2.44	1.45	2	1	5	0.53	−1.15	0.64
9	1.59	1.05	1	1	5	1.79	2.36	0.56

*Note: r* = item/total polyserial correlation.

### 3.1 Confirmatory Factor Analysis (CFA)

We tested two alternative models, a single‐factor model (CFA01) and a correlated two‐factor model (CFA02). Both models presented a very good fit, had higher loadings with their respective fit. The standardised loadings for the latent factor were as follows: 0.741 for item 1, 0.820 for item 2, 0.764 for item 3, 0.838 for item 4, 0.810 for item 5, 0.686 for item 6, 0.737 for item 7, 0.718 for item 8, and 0.800 for item 9, respectively (see also Table 2). However, the latent correlation between the two factors in the second model (CFA02), was extremely high (0.99), making the two factors hardly distinguishable from a statistical point of view. Also, the single‐factor model is more parsimonious and should be preferred compared to the two‐factor model, *Δχ* ^*2*^(1) = 0.212, *p* = 0.645 (the more parsimonious model is chosen when the *p*‐value is not statistically significant). Notably, that the two‐factor model provided a very good fit, which was almost identical to the one‐factor model. The choice of the best model was based on a parsimony criterion rather than a lack of fit in either model.

**TABLE 2:** Model fit statistics.

	*χ* ^2^	df	*p*	CFI	TLI	RMSEA	SRMR
CFA							
CFA01	47.98	27	0.008	0.988	0.983	0.068	0.049
CFA02	47.89	26	0.006	0.987	0.982	0.071	0.049
CFA03	47.88	25	0.004	0.986	0.980	0.074	0.049
MGCFA							
MGCFA01	54.31	40	0.065	0.990	0.986	0.065	0.058
MGCFA02	54.77	47	0.204	0.995	0.994	0.044	0.063
MGCFA03	84.68	70	0.111	0.990	0.992	0.050	0.060

Abbreviations: CFA, confirmatory factor analyses; CFI, Comparative Fit Index; MGCFA, multigroup CFA; RMSEA, root mean square error of approximation; SRMR, standardised root mean square residual; TLI, tucker‐lewis index.

A third model (CFA03) was also tested with Item 5 loading on both factors (e.g., Cipora et al. 2015; Schillinger et al. 2018). An explanation may arise from looking at the wording of item 5, which refers to difficult math homework being assigned for the next upcoming class. Such a model, in the current Japanese sample (Table 2), was not statistically superior compared to the one‐factor model, Δχ^2^(2) = 1.267, *p* = 0.531.

### 3.2 Reliability

Reliability was calculated for the single‐factor model using Cronbach's alpha (0.88), alpha for ordered indicators (0.93), and McDonald's *ω* = 0.90; all values were extremely high, meaning that the scale is reliable from a statistical point of view. We also tested test–retest reliability, at one month distance, which was also very good, *n* = 169, *r* (167) = 0.72, *p* < 0.001.

### 3.3 Validity

Having established that the scale has good reliability, we proceeded to test various forms of validity. In terms of the convergent validity, the correlation with the J‐MAS was very good, *n* = 169, *r* (167) = 0.83, *p* < 0.001, meaning that the two constructs are highly related to each other, and are measuring the same construct.

As for the concurrent validity, the relationship between the AMAS and the Japanese math test was statistically significant and moderate in terms of magnitude, *r* (167) = −0.28, *p* < 0.001. We also calculated the concurrent validity of the J‐MAS, *r* (167) = −0.23, *p* = 0.003. In fact, the two scales (i.e., the AMAS and the J‐MAS) were not statistically different from each other, ZH = −1.20, *p* = 0.231, in terms of their predictive power over the Japanese math test.

### 3.4 Analyses on Gender

Having established that the AMAS presents good reliability and validity values, it was also important to demonstrate that the test is invariant between boys and girls. For these reasons, a multigroup analysis was implemented, testing several progressively stricter forms of invariance. One assumption of the ordered analysis we used was that there should not be any empty cells in the data set. This means that for a questionnaire with a 5‐point scale ranging from 1 to 5, calculations for each item are performed only if data are available for each point on the scale (i.e., at least one participant for each level of the scale on every item). A preliminary check showed that in the group of boys, no children had a response of 5 on Item 7. Therefore, Item 7 was excluded from the analyses.

In the first model (MGCFA01), the same structure was imposed on both groups, and the fit was excellent (Table 2). We then tested stricter forms of invariance. In the second model (MGCFA02), equality of the loadings was imposed; again, the fit was excellent (Table 2), and the model was comparable to the previous one, Δχ^2^ (7) = 5.22, *p* = 0.633, ΔCFI = −0.004, indicating that this model should be preferred. In the third model (MGCFA03), equality of thresholds was imposed; the fit was again excellent (Table 2), and this model was comparable to the previous one, Δχ^2^ (23) = 30.96, *p* = 0.124, ΔCFI = 0.005, suggesting that this model should also be preferred.

### 3.5 Analyses on Age

Descriptive statistics revealed that, overall, girls exhibited higher levels of math anxiety, while simultaneously performing better on the math test. Additionally, second‐grade students showed higher levels of math anxiety compared to fifth‐grade students (Table 3). We also conducted a series of MGCFA analyses on younger and older children confirmed partial scale invariance between the two groups. Detailed results of these analyses are provided in the Table S01.

**TABLE 3:** Descriptive statistics for the AMAS and the Japanese math test.

Group	*N*	*M*	SD
AMAS			
Boys	103	18.47	9.25
Girls	66	22.80	9.15
2nd graders	93	18.41	8.51
5th graders	74	21.69	9.49
2nd graders boys	58	17.0	9.13
2nd graders girls	37	22	8.72
5th graders boys	45	20.3	9.18
5th graders girls	29	23.8	9.74
Japanese math test			
Boys	103	−0.11	1.18
Girls	66	0.18	0.99

*Note*: Japanese math test scores are in standardised values.

## 4 Discussion

The aim of the present study was to test the applicability of the Japanese version of the AMAS to a sample of elementary‐school children. The results showed that the scale has good psychometric properties, including good reliability and validity. Additionally, the scale was found to be invariant between boys and girls and showed partial invariance between younger and older children. Interestingly, the results also indicated that girls, despite having higher anxiety scores, performed similarly to boys and were slightly superior in the math test Table 3.

Concerning the factorial structure of the AMAS, we found that the scale was better represented using a single‐factor structure. This finding is not entirely consistent with the two factors found in the original standardisation (Hopko et al. 2003) and other adaptations of the scale (Caviola et al. 2017; Martín‐Puga et al. 2022; Milovanović and Branovački 2021; Sadiković, Milovanović, and Oljača 2018; Szczygieł 2019). Notably, only a few adaptations of the AMAS scale have statistically tested the two‐factor structure against the one‐factor structure (e.g., Caviola et al. 2017; Vahedi and Farrokhi 2011), while in other cases, this was not tested (e.g., Carey et al. 2017; Cipora et al. 2015). Notably, the fit differences between the one‐factor and two‐factor models in this paper were minimal. This suggests that the underlying structure for Japanese children in the AMAS is not incompatible with the two‐factor solution found in other adaptations.

Some studies reported inconsistent factor structures. For example, some studies reported a model involving a correlated two‐factor structure, with Item 5 loading on both factors (e.g., Cipora et al. 2015; Schillinger et al. 2018). An explanation may arise from the wording of Item 5, which refers to difficult math homework being assigned for the next class. In the current Japanese sample, such a model was not statistically superior to the one‐factor model. These results suggest that the level of anxiety elicited by this item may vary significantly according to school systems. For instance, in a more structured educational system like Japan, this item could elicit test anxiety since the schoolwork will be graded and reviewed by teachers. In other educational systems, this item might elicit only mild learning anxiety. Thus, the variability in the factor structure of the AMAS may reflect not only the perceived level of anxiety but also be closely linked to contextual factors such as school readiness, classroom climate and teaching expectations (Johnston, Wildy, and Shand 2022; Ren and Smith 2018; Wang and Degol 2016).

It is also worth noting that, to the best of our knowledge, the total score, and not the two‐factor scores, is typically used (e.g., Delage et al. 2022; Mammarella, Caviola, et al. 2018; Mammarella, Donolato, et al. 2018). Consequently, the different predictive value of the two‐factor version was never established, implying that the shared variance across the two factors explains most of the variance in mathematics, supporting the use of a single factor. This single‐factor structure aligns with the idea of an underlying general MA factor encompassing all other dimensions. In fact, the two factors observed in other studies seem to be highly correlated, which might indicate the presence of a latent general anxiety factor.

The scale also presents good psychometric properties. Concerning reliability, results showed that measures of internal consistency are very good, as is the test–retest reliability. The scale is strongly correlated and demonstrates similar predictive power compared to the J‐MAS, which is arguably longer and therefore time‐consuming. In terms of concurrent validity, the relationship between the AMAS and the math test (i.e., −0.28) was close to the meta‐analytic value (i.e., −0.30) found by Caviola et al. (2022), confirming the scale's good predictive value. The adaptation of a widely internationally used MA scale will also facilitate the comparison of Japanese children with children from other countries in cross‐cultural studies.

The results from the multigroup analyses on boys and girls are particularly interesting. We found that the scale was invariant across boys and girls, indicating a similar functioning in both groups. Notably, girls exhibited higher scores on the scale compared to boys. Despite higher levels of MA, girls performed equally well, or even better, in mathematics compared to boys (see Satake and Amato 1995 for similar results). This finding is noteworthy, as it suggests that girls can perform as well as boys in mathematics despite having higher MA, corroborating results from other Japanese (e.g., Satake and Amato 1995) and Western samples (e.g., Hill et al. 2016). Previous studies have found that girls are underrepresented in STEM departments (e.g., in physics), which is inconsistent with their good performance in math (Ikkatai et al. 2021). It is also noteworthy that among high‐performing students in mathematics or science, fewer than one in ten boys in Japan are expected to work as science or engineering professionals by the age of thirty, while only about one in thirty girls are expected to do so (OECD 2018). The causes of these differences are not entirely clear, but higher levels of MA might play a key role in explaining this phenomenon.

Data from various international evaluations, such as PISA and TIMSS, indicate that general anxiety levels amongst Japanese individuals are not excessive and align with those observed in many other countries (Lau et al. 2022). However, Lee (2009), analysing PISA 2003 data, reported relatively high anxiety levels amongst 15‐year‐old Japanese students. Similarly, Stankov (2010), building on Lee (2009) data, suggested that Confucian countries, including Japan, exhibit higher anxiety levels. Notably, this conclusion was drawn from a single survey of secondary‐grade students. In fact, this focus on older children complicates direct comparisons with our findings.

Our analysis of Japanese children on the AMAS found that anxiety levels were not excessive. The younger age of our sample compared to other standardisation groups may partly explain this result. Evidence also suggests that younger Japanese children are not necessarily more anxious than their Western counterparts. For example, a cross‐cultural comparison found that Japanese adolescents reported significantly lower anxiety symptoms than English adolescents, highlighting the potential influence of cultural differences in early learning experiences and educational environments (Essau, Ishikawa, and Sasagawa 2011). Similarly, a study of Japanese, Chinese and American high school students revealed that Japanese students experienced lower anxiety levels than their American peers, which was attributed to the supportive and less punitive nature of Japan's educational system (Crystal et al. 1994). The Japanese primary school environment is particularly child‐oriented, with frequent breaks and an emphasis on encouraging questions, fostering a less stressful learning atmosphere (Satake and Amato 1995). Consistent with this, Satake and Amato (1995) reported that Japanese fifth and sixth graders exhibited lower anxiety levels compared to their North American counterparts. These findings suggest that Japanese educational practices and parenting styles may contribute to reduced anxiety in academic settings, particularly during primary education.

An intriguing study by Oie and Fujii (2017) longitudinally assessed different cohorts of Japanese students on MA, finding that MA levels tended to be higher in older students. Our re‐analysis of their original data confirmed a statistically significant difference between the fifth and eighth graders at Time 1 (Cohen's *d* = −0.454, 95% CI [−0.606, −0.302]). In addition, Satake and Amato (1995) standardised the MAR‐E scale in a sample of fifth‐ and sixth‐grade Japanese children, confirming that girls exhibited higher anxiety levels than boys. This finding may partially support the notion that older children tend to be more anxious than younger ones, and this is particularly true for girls. It is also worth noting that the factorial structure observed in Japanese children in the study by Oie and Fujii (2017) differed somewhat from that observed in North American children, suggesting the possibility of cross‐cultural differences influenced by distinct cultural and environmental contexts. This finding is intriguing and may partially explain why a single‐factor structure was more appropriate for our sample, whereas a two‐factor structure remains valid in Western countries.

Despite presenting very interesting findings, the current study has some limitations that should be recognised. We believe that our results should be replicated with a larger sample, including older children, adolescents and adults. Our sample was a convenience sample, and we had limited access to schools, which restricted our ability to test a larger number of participants. Expanding the sample size would allow us to investigate whether differences in MA increase over time. Additionally, it is crucial to conduct longitudinal studies to evaluate changes in the same group of children over time. This approach is particularly important as most research on this topic relies on cross‐sectional samples. Longitudinal studies would provide insights into the developmental trajectory of MA and its potential long‐term effects. Furthermore, these results should be expanded to include clinical samples to determine if this scale can be used effectively in clinical settings, alongside more comprehensive scales. Such studies would help ascertain the scale's utility in identifying MA in children with clinical levels of anxiety.

## 5 Conclusions

Our results showed that the Japanese version of the AMAS scale is reliable, valid and invariant between genders. We believe that this adaptation of the AMAS can contribute significantly to the existing literature, demonstrating that girls, despite exhibiting higher MA, do not differ in terms of mathematical performance (Hill et al. 2016). Additionally, we believe there are clinical implications, as the AMAS is a short yet highly valid and reliable instrument that can be used as a screening measure for MA in young children.

## Ethics Statement

The study was conducted in accordance with the Declaration of Helsinki and approved by the Ethics Committee of Joetsu University of Education.

## Consent

Assent and informed consent were obtained from all participants and their parents or legal guardians, respectively, prior to their participation.

## Conflicts of Interest

The authors declare no conflicts of interest.

## Supporting information




**Data S1.** Supporting Information.
**Table S01.** Model fit statistics.

## Data Availability

The participants of this study did not give written consent for their data to be shared publicly, so due to the sensitive nature of the research is not available.
