# Supplementary material for: The Abbreviated Math Anxiety Scale (AMAS): Applicability and Utility in a Sample of Japanese Elementary School Children
Source: Int J Psychol. 2025 Feb 11;60(2):e70015. doi: 10.1002/ijop.70015 (PMC11813552; doi:10.1002/ijop.70015)
Supplement: Supplementary file 1 — Data S1. Supporting Information. Table S01. Model fit statistics. [file IJOP-60-e70015-s001.docx]

**The Abbreviated Math Anxiety Scale (AMAS): Applicability and Utility in a Sample of Japanese Elementary School Children.**

**Supplementary Online Information**

**MCCFA on younger and older children**

We also tested several steps of invariance for younger and older children. For these reasons, a multigroup analysis was implemented, testing several progressively stricter forms of invariance. In the first model (MGCFAS01), the same structure was imposed on the two groups, the fit was acceptable (Table S01), and we decided to test stricter forms of invariance. In a second model (MGCFA02), equality of the loadings was imposed, in this case the fit decreased considerably, *Δχ^2^*(8) = 37.102, *p* < .001, *ΔCFI* = .021 (Table S01). We therefore decided to free the loadings of two items (items 1 and 6) testing a partial invariant model (MGCFAS02^p^); the fit in this case the fit was good (Table S01), and this model was comparable with the first one only testing the structure, *Δχ^2^*(6) = 7.391, *p* = .286, *ΔCFI* = -.001, meaning that this model should be preferred. This model showed partial invariance between the two groups, with loadings on the first item being larger in the older children and loadings on the sixth item being larger in the younger children. This might reflect the fact that using tables is more related to math anxiety in older children because the teaching becomes more structured, and the operations get more difficult. On the other hand, listening to a lecture might be more related to anxiety in younger children, who might feel more anxious in the presence of the teacher. In contrast, older children, having had more experience and the same teacher for a longer period of time, might find this item less related to math anxiety. These differences likely reflect genuine differences between the age groups, but the overall structure of the questionnaire seems satisfactory. In a third model (MGCFAS03), equality of thresholds was also imposed, also in this case the fit was good (Table S01), and this model was comparable to the previous one, *Δχ^2^*(26) = 30.233, *p* = .258, *ΔCFI* = .002, here again this model should be preferred.

Table S01

*Model fit statistics*

|  | *χ*^2^ | *df* | *p* | CFI | TLI | RMSEA | SRMR |
| --- | --- | --- | --- | --- | --- | --- | --- |
| MGCFA |  |  |  |  |  |  |  |
| MGCFAS01 | 76.10 | 54 | .025 | .989 | .986 | .070 | .064 |
| MGCFAS02 | 129.60 | 62 | .000 | .968 | .963 | .114 | .101 |
| MGCFAS02^p^ | 80.32 | 60 | .041 | .990 | .988 | .064 | .072 |
| MGCFAS03 | 111.39 | 86 | .034 | .988 | .990 | .059 | .066 |

*Note*. CFI = Comparative Fit Index; TLI = Tucker-Lewis Index; RMSEA = Root Mean Square Error of Approximation; SRMR = Standardized Root Mean Square Residual; CFA = Confirmatory Factor Analyses; MGCFA = multigroup CFA.

MGCFA02^p^ Partial invariant model
